# Learning from Fifteen Years of Genome-Wide Association Studies in Age-Related Macular Degeneration

**DOI:** 10.3390/cells9102267

**Published:** 2020-10-10

**Authors:** Tobias Strunz, Christina Kiel, Bastian L. Sauerbeck, Bernhard H. F. Weber

**Affiliations:** 1 Institute of Human Genetics, University of Regensburg, 93053 Regensburg, Germany; Tobias.Strunz@klinik.uni-regensburg.de (T.S.); Christina.Kiel@klinik.uni-regensburg.de (C.K.); Bastian.Sauerbeck@klinik.uni-regensburg.de (B.L.S.); 2 Institute of Clinical Human Genetics, University Hospital Regensburg, 93053 Regensburg, Germany

**Keywords:** age-related macular degeneration, GWAS, eQTL, TWAS, analytical review

## Abstract

Over the last 15 years, genome-wide association studies (GWAS) have greatly advanced our understanding of the genetic landscape of complex phenotypes. Nevertheless, causal interpretations of GWAS data are challenging but crucial to understand underlying mechanisms and pathologies. In this review, we explore to what extend the research community follows up on GWAS data. We have traced the scientific activities responding to the two largest GWAS conducted on age-related macular degeneration (AMD) so far. Altogether 703 articles were manually categorized according to their study type. This demonstrates that follow-up studies mainly involve “Review articles” (33%) or “Genetic association studies” (33%), while 19% of publications report on findings from experimental work. It is striking to note that only three of 16 AMD-associated loci described de novo in 2016 were examined in the four-year follow-up period after publication. A comparative analysis of five studies on gene expression regulation in AMD-associated loci revealed consistent gene candidates for 15 of these loci. Our random survey highlights the fact that functional follow-up studies on GWAS results are still in its early stages hampering a significant refinement of the vast association data and thus a more accurate insight into mechanisms and pathways.

## 1. Introduction

Age-related macular degeneration (AMD) is the most common cause of visual impairment and blindness in industrialized countries [1]. The clinical phenotype is progressive with age and is characterized by degenerative processes and ultimately the loss of photoreceptor cells. The natural history of the disease is mainly attributable to a dysregulation of the retinal support system, dependent on the mono-layered retinal pigment epithelium (RPE), Bruch’s membrane, and the choriocapillaris/choroid blood supply [2]. While a number of studies have shown that AMD patients exhibit changes in blood serum components [3,4], the exact mechanisms of disease development are still not fully understood and treatment options are limited.

In addition to environmental factors, genetic predisposition plays a crucial role in AMD susceptibility rendering the disease a complex disorder. A twin study of Seddon et al. (2005) estimated the genetic contribution to be as high as 71% [5]. A breakthrough in AMD genetics, as for complex diseases in general, was achieved by a first successful genome-wide association study (GWAS) conducted in 2005. Klein et al. (2005) compared the genotypes of 96 AMD patients and 50 controls and identified genetic variants around the Complement Factor H (*CFH*) gene locus to be reproducibly associated with AMD [6]. This initial study was followed by numerous GWAS for AMD, which included increasing numbers of individual DNA samples consequently revealing additional AMD-associated loci [7]. The most recent and largest AMD GWAS was published in 2016 by the International AMD Genomics Consortium (IAMDGC) and included 16,144 patients and 17,832 controls. Altogether, 52 independent genetic signals distributed over 34 loci showed an association with AMD at genome-wide significance [8].

From the beginning, GWAS have produced a huge pool of data for a plethora of complex traits and diseases. In consequence, questions of what can be accomplished with these vast data sets and how to gain functional insights from the many linked association signals at a single locus became increasingly prominent. Especially, disease-associated genetic variants in non-coding regions of the genome tenaciously resist to offer a simple interpretation of their functionality [9,10]. Last but not least, other biological and statistical constraints, such as linkage disequilibrium (LD) or necessary statistical adjustments for a high number of conducted tests, further complicate data interpretation and consequently the identification of disease-relevant genes.

Initially, potential candidate genes underlying the genetic association signals within a locus were chosen simply by proximity to the respective lead genetic variant. More intelligent strategies were sought, as increasing evidence emerged that non-coding variants can have far-reaching effects [11]. Clever statistical approaches were designed to fine-map loci or to identify the causative genetic signal, e.g., by using Mendelian randomization [12,13]. The identification of pleiotropic genetic signals highlighted potentially shared pathways between complex diseases and traits [14]. More recently, technological advances enabled the large-scale analysis of molecular phenotypes like mRNA abundance or DNA methylation. Studying gene expression regulation in dependence of genetic variation generates data known as expression quantitative trait loci (eQTL) [15,16]. eQTL studies aim to uncover direct mechanisms of gene expression regulation and their results are likely to suggest candidate genes involved in disease pathology. Advances in machine learning approaches may facilitate an even more global analysis of gene expression regulation. This latter methodology is not only focused on individual genetic variants but instead integrates GWAS and gene expression datasets to identify gene–trait associations in so-called transcriptome-wide association studies (TWAS) [17,18].

In this review, we follow up on the two largest GWAS conducted on AMD so far and explore in an exemplary manner, to what extend the obtained association data were processed in the following years to gain information on biological mechanisms underlying the disease pathology. We further focus on current eQTL and TWAS studies related to AMD, which are becoming more popular. Our aim is to present a critical assessment of how the available large-scale AMD GWAS data are exploited to identify and evaluate potential disease-related candidate genes for future research into AMD etiology.

## 2. Materials and Methods

### 2.1. Curation of Articles Citing the Two AMD GWAS Fritsche et al. 2013 and Fritsche et al. 2016

The Web of Science Core Collection [19] was searched for articles citing the AMD GWAS articles published by Fritsche et al. 2013 [20] and/or Fritsche et al. 2016 [8] (Figure S1). The publications were manually appraised based on abstract and full text content, the latter if available. Articles were then assigned to one of five categories: (1) “Genetic association study”, (2) “Experimental study”, (3) “Clinical study”, (4) “Review article”, or (5) “Referencing only”. The category “Genetic association study” refers to follow-up investigations pursuing genetic associations of AMD-related genotypes with any phenotype. Articles that reported any derivative experimental work in continuation of the AMD GWAS results as given in references [20] and/or [8] were assigned to the category “Experimental study”. “Clinical study” is a category focusing on clinical phenotype-associated issues but not on the genetics of AMD. ”Review articles” aggregate reviews, editorial material and comments based on the two publications [8,20]. Articles, which cited references [8,20] but only referred to AMD in general terms were assigned to the category “Referencing only”. We removed articles listed multiple times in the Web of Science citation report. Further, articles citing both AMD GWAS [8,20] were only considered in the evaluation of Fritsche et al. 2016 [8] citing articles. Multi-assignment of categories was allowed for the categories “Genetic association study” and “Experimental study”.

### 2.2. Locus Analysis of 34 Known AMD-Associated Loci

All publications initially categorized into “Genetic association study” or “Experimental study” were re-reviewed individually to determine if they investigated defined loci or if they rather pursued a more general approach. Next, the respective loci were considered for their overlap with the 34 AMD-associated loci in the GWAS published in [8]. Studies replicating only defined GWAS associations in additional populations were not considered in this analysis. Loci, which were investigated by a genetic association study and additional experiments in the same article were counted only once in one of the two categories.

### 2.3. Curation and Quality Control of Studies Investigating AMD in the Context of Gene Expression Regulation

PubMed [21] was searched for the term “Age-related macular degeneration” with the combinations “gene expression regulation”, “eQTL”, or “TWAS”. Our study inclusion criteria filtered for studies globally investigating the transcriptome in at least 70 samples. The literature search identified six potentially relevant studies of which five were considered for further evaluation (Table 1). Wang et al. (2019) [22] were excluded as this study merged eQTL data from 44 tissues (GTEx v6 [23]) and only considered the most significant eQTL *p*-Value per variant throughout all tissues which is not in-line with generally accepted eQTL or TWAS concepts [17,18,24].

Next, we extracted AMD-related significant results of the remaining five studies. As each study used a different adjustment protocol for multiple testing, the significance thresholds as specified in the respective study were maintained. The most stringent cutoff was chosen, if a study applied several significance thresholds. We further adjusted effect directions according to the AMD risk increasing allele to allow a uniform comparison of findings across the five studies. Study-specific parameters were as follows:

Ratnapriya et al. (2019) [25] conducted an eQTL and a TWAS analysis based on 406 samples. Significant eQTL were identified using a two-step protocol. First, permutations were conducted to identify a gene-specific significance threshold. This threshold was then adjusted for multiple testing across all genes using the false-discovery rate (FDR) from Storey et al. (2003) [26] at 0.05. We downloaded the eQTL results [27] which were then filtered for the 52 AMD-associated lead variants [8]. In their TWAS analysis, Ratnapriya et al. (2019) chose a Bonferroni correction for multiple testing (threshold 0.05) and required the genetic expression model R2 to be at least 0.01 [25].

Orozco et al. (2020) [28] investigated eQTL in a total of 121 samples from retinal and combined RPE and choroid (RPE/choroid) tissue. Further, they distinguished between macular and non-macular tissue and applied a Benjamini and Hochberg FDR threshold of 0.05 [29]. As not all summary statistics are available for download, we only considered the significant AMD loci provided in Data S4 by Orozco et al. (2020). The eye-eQTL database [30] containing the eQTL data of Orozco et al. (2020) was used to manually obtain the eQTL effect alleles.

Our group published three studies with regard to gene expression regulation in the context of AMD genetics. First, a mega-analysis of eQTL in 588 liver samples and a Benjamini and Hochberg FDR threshold of 0.05 to identify significant eQTL of AMD lead variants [31]. Second, a mega-analysis of eQTL in 311 retinal tissues, which also included the healthy donor samples of Ratnapriya et al. (2019) [32]. The correction for multiple testing was done as described for Ratnapriya et al. (2019). Third, a TWAS based on 27 tissues from GTEx and the individual genotypes of all samples from the IAMDGC with European ethnicity [8,33]. AMD-associated genes were identified separately for each tissue and adjustment for multiple testing used a Benjamini and Hochberg FDR threshold of 0.001.

## 3. Results

### 3.1. Investigations Following the AMD GWAS of Fritsche et al. 2016

In a first step, we aimed to investigate how the research community further processed the data given in the most recent comprehensive AMD GWAS published by Fritsche et al. in 2016 [8] (Figure S1). We identified 366 studies citing the corresponding publication and categorized the follow-up activities in five subject groups (Table S1 and Figure S2). Interestingly, the two most frequent responses to reference [8] fall into the categories “Genetic association studies” (113 of 366; 30.9%) and “Review articles” (113 of 366; 30.9%). In contrast, only 66 of 366 (18.0%) of the follow-up publications reported on experimental work to investigate functional implications of AMD-associated variants.

**Figure 1:**
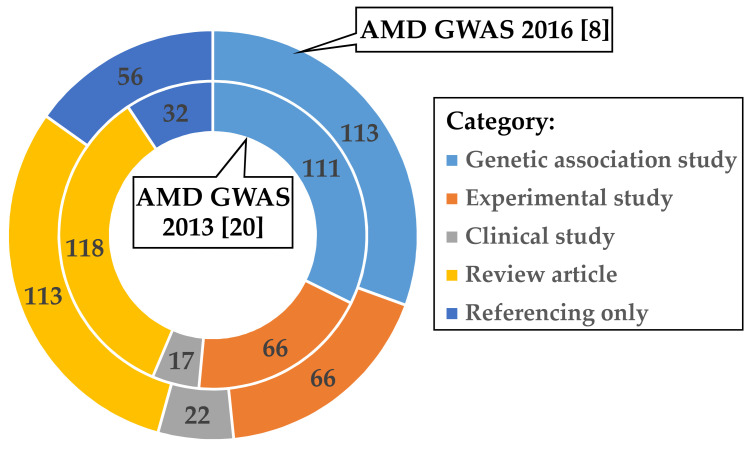
Categorized responses of published work citing the AMD GWAS of Fritsche et al. 2013 [20] and Fritsche et al. 2016 [8]. After quality control (see Methods), 337 publications cited Fritsche et al. (2013) (inner circle) [20] during the period 2013–2020 and 366 publications referred to Fritsche et al. (2016) (outer circle) [8] during the period 2016–2020. Articles were manually assigned to one of the five categories: “Genetic association study” (light blue), “Experimental study” (orange), “Clinical study” (grey), “Review article” (yellow), or “Referencing only” (dark blue). Articles citing both AMD GWAS [8,20] were only included in the Fritsche et al. 2016 [8] evaluation. Multi-assignment of categories was allowed for the categories “Genetic association study” and “Experimental study”. This was the case for seven studies citing Fritsche et al. 2013 [20] and four studies citing Fritsche et al. 2016 [8] (Tables S1 and S2).

To evaluate if these findings could be biased towards reference [8], we analyzed in an identical fashion the citation records available for the previous AMD GWAS published by Fritsche et al. in 2013 [20]. After removing 107 publications which referred to both GWAS [8,20], 337 articles remained for further categorization (Table S2 and Figure S3). Remarkably, the distribution of follow-up activities within the subject groups is highly comparable between the two AMD GWAS reports although the time period between the publication dates of the two studies differed by about three years (Figure 1). Over 35% (118 of 337) of publications citing Fritsche et al. 2013 [20] were “Review articles”, followed by “Genetic association studies” (111 of 337; 32.9%) and “Experimental studies” (66 of 337; 19.6%).

### 3.2. Investigation of Defined Loci Based on the Reference Data of Fritsche et al. 2016

**Figure 2:**
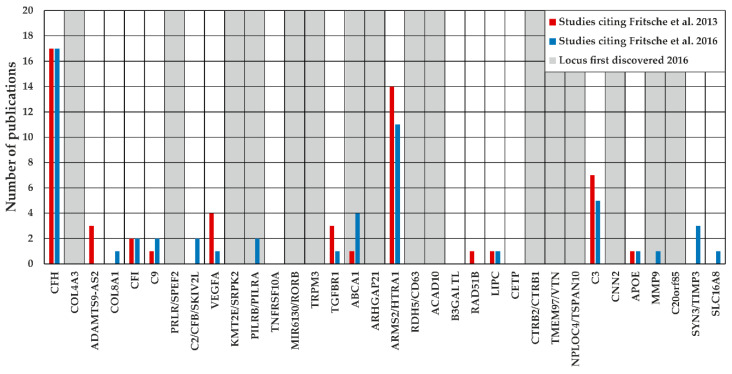
Investigated loci of studies citing the AMD GWAS of Fritsche et al. 2013 [20] and Fritsche et al. 2016 [8] Forty three publications referred to Fritsche et al. 2013 (red) [20] and analyzed genomic regions, which are known to be AMD-associated. In addition, 11 of these studies investigated multiple loci resulting in 55 extensively analyzed genomic regions. Similarly, 38 publications referring to Fritsche et al. 2016 (blue) [8] analyzed an AMD-associated locus with 12 articles reporting findings regarding multiple loci. Altogether 55 loci were investigated by studies citing Fritsche et al. 2016 [8]. AMD-associated loci which reached genome-wide significance in the AMD GWAS of Fritsche et al. 2016 [8] for the first time are highlighted in grey shading.

Next, we focused on the question whether publications citing the AMD GWAS of Fritsche et al. 2013 [20] or 2016 [8] addressed a defined genomic region. The latest GWAS of Fritsche et al. 2016 reported 34 loci to be AMD-associated with genome-wide significance [8], with these loci serving as reference in our analysis. Referring to Fritsche et al. 2013 [20], a total of 74 publications assigned topically to “Genetic association study” or “Experimental study” investigated a specific locus, whereas this was the case for 49 studies citing Fritsche et al. 2016 [8]. Remarkably, not all analyzed loci harbored variants associated with AMD at genome-wide significance, which results in 58.1% (43/74, Fritsche et al. 2013), respectively 77.6% (38/49, Fritsche et al. 2016) of studies for the analysis of AMD-associated loci. Furthermore, several studies investigated more than one locus, resulting in 55 investigated loci by studies citing Fritsche et al. 2013 [20] and 55 loci for studies referring to Fritsche et al. 2016 [8] (Figure 2).

The AMD GWAS of Fritsche et al. 2016 identified 34 AMD-associated loci of which 16 reached genome-wide significance for the first time. Remarkably, only three of these “novel” loci (see “*PILRB*/*PILRA*”, “*ABCA1*”, and “*MMP9*”) were investigated further in the four years after publication (Jan 2016–Jul 2020). In contrast, 14 of the 18 loci already known to be AMD-associated before 2016, were studied and reported in at least one publication. Strikingly, most of the studies focused on loci “*CFH*” and “*ARMS2*/*HTRA1*”. Altogether, these observations emphasize that the AMD GWAS sparsely triggered locus-specific experiments. This is particularly true regarding loci identified as genome-wide associated AMD signals for the first time in 2016.

### 3.3. AMD Genetics and Gene Expression Regulation

Large scale eQTL and TWAS studies are suited to elucidate the potential influence(s) of disease-associated genetic variation on gene expression regulation. Our literature search identified five of these studies with suitable data that considered the results of Fritsche et al. 2016 in their analyses (Table 1). Ratnapriya et al. (2019) were the first to perform eQTL and TWAS analyses in 406 retinal samples [25]. This was followed by a study of Orozco et al. (2020) that calculated eQTL in 121 retinae and additionally in RPE/choroid samples [28]. Both studies included, apart from control retinae, additionally AMD patient samples to a variable extend. Strunz et al. (2020) performed an eQTL mega-analysis using three datasets based on 311 retinal tissue samples of exclusively healthy donor eyes, including 94 control samples of Ratnapriya et al. (2019) [25,32]. Two further studies targeted gene expression regulation in extraocular tissues including 588 liver tissue samples from four independent studies [31], and various tissues originating from the GTEx database as well as individual genotype data from the IAMDGC sample set [8,33] (Table 1).

The eQTL/TWAS studies identified genetically regulated genes in several AMD-associated loci (Table 1 and Table S3). Strunz et al. (2018) reported eQTL findings from liver tissue in five AMD-associated loci [31]. In retinal tissue, the findings varied from significant correlations in four [25,32] to eleven [28] AMD loci. This variability is not likely to just emerge from methodological differences as e.g., all studies imputed their genotype data from the 1000 Genomes project reference panel [34]. The number of effects identified does not correlate with sample size, as exemplified in the GTEx project [24].

### 3.4. Gene Expression Regulation is Likely Associated with 15 Known AMD Loci

Findings for gene expression regulation in retinal tissue appear to be variable and highly study dependent. Here, we develop a strategy to identify robust and tissue-related effects. For retinal tissue, we define four categories of genes regulated by AMD-associated genetic variants. Category 1 comprises effects found in at least two retinal studies and in more than two additional tissues other than retina. Categories 2 and 3 require only one of the thresholds defined in category 1 and focus either on effects present predominantly in retina (category 2) or in other tissues (category 3). Category 4 summarizes effects, which either failed to replicate in several retinal datasets or were found in less than three tissues. The latter threshold was set arbitrarily to enable a more focused interpretation of regulatory effects. It should be mentioned that there were partially overlapping samples used in the studies, e.g., the mega-analysis by Strunz et al. (2018) [31] included some GTEx samples which were also enrolled in the subsequent TWAS [33].

In eight of the 34 AMD loci identified in reference [8], subsequent studies detected no gene expression regulation by AMD-associated genetic variants. Another eleven loci were assigned to category 4 although no reliable conclusions can be drawn (Table S4). Findings for the remaining 15 known AMD loci allowed classification of the associated genes to categories 1, 2 or 3 (Table 2).

Genetic variants in four known AMD loci (see “*PILRB/PILRA*”, “*ARMS2/HTRA1*”, “*B3GALTL*” and “*TMEM97/VTN*”) reveal effects on gene expression regulation present in retinal and various other tissues (Category 1). Remarkably, with exception of the study by Orozco et al. (2020) [28], an AMD-associated regulation of the genes *PILRA* and *PILRB* was reported in all studies and consistently showed an upregulation with increased AMD risk. Another three loci (see “*CFI*”, “*C2/CFB/SKIV2L*”, and “*RDH5/CD63*”) revealed gene regulatory effects predominantly in retina in comparison to data available from extraocular tissues [31,33] (category 2). The gene *HLA-DQB1* (locus “*C2/CFB/SKIV2L*”) is part of the major histocompatibility complex and was explicitly excluded in the TWAS by Strunz et al. (2020). Consequently, no conclusions about gene expression regulation in extraocular tissues can be drawn [33]. Eight AMD loci (category 3: “*CFH*”, “*COL8A1*”, “*TNFRSF10A*”, “*CETP*”, “*CTRB2/CTRB1*”, “*C3*”, “*CNN2*”, and “*MMP9*”) revealed gene expression regulation effects only in extraocular tissues while five of these harbor multiple genes which were assigned to different categories. The effect directions of gene expression regulation were in most instances (31 of 37 genes in Table 2) consistent across studies and tissues. In contrast, the six genes *HTRA1*, *B3GLCT*, *CFH*, *PLEKHA1*, *BTBD16*, and *SLC12A5* revealed different effect directions by AMD-associated variants. It should be noted, however, that gene expression regulation can be highly tissue-specific [24], thus each effect needs to be considered in a tissue-specific context.

The undirected TWAS approach as reported by Ratnapriya et al. (2019) [25] and Strunz et al. (2020) [33] allows detection of effects outside the 34 known AMD loci [8]. Together, the two studies identified 21 AMD-associated genes distributed over several genomic regions (Table S5). Orozco et al. (2020) contributes an additional locus harboring the potential AMD-associated eQTL gene *TRPM1*. Remarkably, none of the 22 genes overlap in-between the three studies.

Reevaluation of the five studies, all addressing gene expression regulation by genetic variation, revealed a total of 37 reproducible genes distributed over 15 loci with a potential role in AMD pathology. In contrast, due to the high degree of heterogeneity in-between the studies a list of 80 genes was assembled (category 4, Table S4), for which no further information can be derived. The latter conclusion is also the case for genes outside of known AMD-associated loci. Nevertheless, our analysis should offer new starting points to investigate the pathology of AMD in more detail. 

## 4. Discussion

Applications of GWAS approaches date back almost fifteen-years and have a successful history of mapping thousands of loci involved in complex traits and phenotypes. From the outset, AMD played a pioneering role in the realization of initially theoretical principles. This is largely due to the strong genetic effects on disease risk making AMD a perfect model to explore many aspects of complex disorders. The ultimate goal of genetic studies investigating complex diseases is to clarify the underlying biological processes which in turn are thought to initiate the development of targeted and effective therapies. In this analytical review, we critically examined the advances achieved in one of the best studied complex disorders, namely AMD. To this end, we comprehensively analyzed the literature citing the latest two large AMD GWAS, one published in 2013 [20] and one in 2016 [8]. To our astonishment, the majority of follow-up articles referring to the two GWAS regardless of their publication date cover the areas of “Review articles” and “Genetic association studies”. Only a small fraction of studies citing one of the two major GWAS publications [20] focused on continuing experimental work, e.g., to narrow a genomic region of interest or to delineate functional correlations of associated genetic variants. Even more surprising, many of the highly significant AMD-associated loci were rarely or not at all considered for follow-up studies. Such observations could suggest that the complexity of GWAS data hamper its interpretation still today. One approach to overcome such problems and to investigate functional consequences of genetic variation are eQTL and TWAS studies, both approaches providing insight to some extent into the genetic influences of tissue-related gene expression. Still, current eQTL results in retinal tissue are inconsistent making further interpretations challenging.

While GWAS are designed to identify genetic associations with complex traits or diseases, follow-up studies, specifically experimental studies, are mandatory to promote a hypothesis-driven approach for understanding the underlying close association between a locus and a trait. Possibly, a reason for the lack of experimental follow-up studies could stem from the high degree of professional specialization required to succeed in the line of approaches from mapping to locus refinement to candidate gene identification to uncovering the consequences of genetic variability and finally to understand disease mechanisms. For example, the statistical geneticist may not be familiar with the requirements and concerns of the biologist applying a plethora of experimental assays to define consequences of genetic variation. Similarly, the statistical geneticist and the biologist may not be quite knowledgeable about subtleties of the clinical picture and thus may miss important information imperative to interpret in vitro findings. Thus, the clinician is needed to lay the groundwork for correlating mechanisms to phenotype. Vice versa, the clinical ophthalmologist may not be aware of the implications of a specific GWAS result for further recruitment needs. Often genetic studies deal exclusively with haplotype structures and allele frequencies but may not consider subtleties in phenotypic features [13,35,36]. Finally, clinical studies often bear the risk to leave out crucial knowledge of genetics in their interpretations and considerations [37,38,39]. A more user-friendly and concise presentation of data is warranted and first public databases aim to address these needs [40,41]. For instance, the UCSC Genome Browser [42] offers information not only on the genomic context of a variant, but also on a multitude of potentially correlated clinical and molecular phenotypes. It seems reasonable to more comprehensively include specialists with expertise in a multitude of traits and diseases as genetics suggests that pleiotropic effects may play a significant role across complex phenotypes [14,43,44].

Another valuable access to link genetic variation with biological function may be provided by eQTL and TWAS studies. These and similar approaches have the power to suggest candidate genes within GWAS loci and even provide effect directions. Unfortunately, eQTL and TWAS data often tend to be inconclusive in direct interpretation. For example, Strunz et al. (2020) have shown that AMD-associated genetics influence gene expression in multiple tissues, however, it is unclear how a decreased expression of *ZKSCAN1* in the tissue classified as “artery aorta” could be related to AMD pathogenesis [33]. In addition, for many genes/proteins only the general function is known so far, whereby a multitude of tissue-specific and often so far unknown roles are expected [24,41]. The condensation of five eQTL and TWAS studies emphasizes some potentially AMD-related effects on gene expression regulation in 26 out of 34 AMD-associated loci. Remarkably, there is the tendency of a consistent and partially tissue overlapping effect in 15 of these loci (Table 2) which provides excellent candidate genes for further investigations. As current research on AMD appears to be focused on the complement system and lipid metabolism, we specifically wanted to highlight two exemplary loci for which the potential role in AMD pathology is not understood so far. One locus harbors the two genes *PILRA* and *PILRB*. The regulation of these genes appears to overlap with the genetic signal of the AMD-association at 7q22.1 [28]. Both genes/proteins are thought to play a role in the regulation of immune responses through CD99 binding [45]. It is further known that PILRA and PILRB act as antagonists in the PTPN6 pathway [46]. Such an antagonistic function, however, conflicts with reproducible findings in the eQTL studies which suggest that both genes are synchronously up- or downregulated. This could suggest that PILRA and PILRB also play a role in processes that have not yet been defined. A second locus may potentially reveal a retina-specific effect. We specifically noted an AMD-associated upregulation of *BLOC1S1* at 12q13.2. BLOC1S1 is part of the BLOC-1 complex, which is required for the synthesis of lysosome-related organelles like melanosomes [47]. BLOC-1 deficiency leads to the rare Hermansky-Pudlak syndrome, which for example is associated with ocular albinism and bleeding diathesis [48]. On the other hand, BLOC1S1 is a regulatory protein for the mitochondrial acetyltransferase program and alters mitochondrial oxygen consumption [49,50]. The upregulation of *BLOC1S1* in retinal tissue could therefore alter the energy metabolism of photoreceptor cells. In addition, changes of mitochondria function are believed to play a role in the AMD disease process [51]. Following such information *BLOC1S1* may well be a relevant player in AMD pathogenesis.

Although eQTL and TWAS studies point out valuable candidates from GWAS loci for further functional studies, it is still mostly unclear by which mechanisms gene expression regulation could influence disease pathogenesis [52,53]. In general, eQTL studies use RNA information from healthy tissues and thus may not provide accurate information for a disease-related situation when pathological damage has occurred for some time possibly causing subsequent molecular alterations never seen in healthy tissue. In fact, it is conceivable that modifications in gene expression regulation could lead to changes in cellular homeostasis. While minor changes in gene expression may not per se be consequential, it is conceivable that their influence increases in combination with other factors. To this end, several studies have shown that aging has an impact on gene expression and chromatin status [54,55,56]. The potential interaction of aging and eQTL effects is in line with the late onset of complex diseases. Additionally, molecular processes like oxidative stress could enhance an impact on cellular homeostasis [57,58]. Furthermore, changes in gene expression regulation could lead to alterations in the activity of regulatory pathways. This aspect is reinforced by our observation that several genes with regulatory roles concerning the immune system or the cholesterol metabolism are influenced by AMD genetics (Table 2). While exact mechanisms of how changes in gene expression regulation may influence disease pathogenesis are currently elusive, interacting and additive processes are arguable.

In summary, this review exemplifies on the complex AMD disorder that the interpretation and further follow-up of initial GWAS data poses a challenge for the research on complex diseases. The comparatively small proportion of experimental follow-up studies on two major AMD GWAS illustrates that clear target genes are still missing hampering insight into novel pathways and disease mechanisms. On the other hand, studies on gene expression regulation are designed to reveal hidden correlations between genetic variation and gene expression and help to identify potentially relevant gene candidates underlying a defined disease pathology. Clearly, additional studies are needed to refine the results of eQTL and TWAS studies. To this end, methodologies such as massively parallel reporter assays or single-cell RNA sequencing have successfully been applied [59,60,61,62]. Nevertheless, an ever-increasing set of data and information necessitates context and as such the collaboration of experts to connect the threads and to improve our understanding of a complex disease, a prerequisite for systems medicine approaches.

## Figures and Tables

**Table 1:** Studies investigating AMD association data in the context of gene expression regulation.

Study	Study Type	Category	Tissue	Sample Size	AMD Status of Tissue Donors	AMD Loci [8] ^1^ with Findings
Ratnapriya et al. (2019) [25]	eQTL, TWAS	Single study	Retina	406	Non-AMD (94), AMD (312)	eQTL: 9, TWAS: 10
Orozco et al. (2020) [28]	eQTL	Single study	Retina, RPE/choroid	121	Non-AMD (98), AMD (23)	11 in retina, 9 in RPE/choroid
Strunz et al. (2020) [32]	eQTL	Mega-analysis	Retina	311	Non-AMD	4
Strunz et al. (2018) [31]	eQTL	Mega-analysis	Liver	588	Unknown	5
Strunz et al. (2020) [33]	TWAS	-	27 tissues	134–421	Unknown	25

^1^ Referring to 34 AMD-associated loci with genome-wide significance identified by Fritsche et al. 2016 [8]; eQTL: expression quantitative trait locus; RPE: retinal pigment epithelium; TWAS: transcriptome-wide association study.

**Table 2:** Known AMD-associated loci [8] harboring gene expression regulatory effects.

Locus ID^1^	Category 1 (Retina + Other Tissues)	Category 2 (Retina)	Category 3 (Predominantly Other Tissues)
*CFH*	-	-	*KCNT2* (−), *CFH* (+/−), *CFHR1* (+), *CFHR3* (+), *ZBTB41*(+)
*COL8A1*	-	-	*NIT2* (−), *TBC1D23* (−)
*CFI*	-	*CFI* (−)	*PLA2G12A* (+), *CASP6* (+)
*C2/CFB/SKIV2L*	-	*HLA-DQB1* (−) ^2^	-
*PILRB/PILRA*	*PILRA* (+), *PILRB* (+), *STAG3L5P* (+)	-	*PMS2P1* (−), *TSC22D4* (+), *ZCWPW1* (+), *NYAP1* (−)
*TNFRSF10A*	-	-	*TNFRSF10A* (−)
*ARMS2/HTRA1*	*HTRA1* (+/−)	-	*PLEKHA1* (+/−), *ARMS2* (−), *BTBD16* (+/−), *DMBT1* (−)
*RDH5/CD63*	-	*BLOC1S1* (+), *AC009779.3* (−)	*RDH5* (−)
*B3GALTL*	*B3GLCT* (+/−)	-	-
*CETP*	-	-	*CETP* (−)
*CTRB2/CTRB1*	-	-	*CFDP1* (−)
*TMEM97/VTN*	*TMEM199* (+)	-	*POLDIP2* (+)
*C3*	-	-	*GPR108* (+)
*CNN2*	-	-	*MED16* (+)
*MMP9*	-	-	*PLTP* (+), *SLC12A5* (+/−)

^1^ Locus ID referring to Fritsche et al. 2016 [8]; ^2^ Region not covered in TWAS by Strunz et al. (2020) [33]; Gene expression is up- (+) or down- (−) regulated by AMD risk variants.

## Supplementary Materials

The following are available online at https://www.mdpi.com/2073-4409/9/10/2267/s1, Figure S1. Workflow overview of literature search and follow-up investigations, Figure S2. Categorization flow-chart of articles citing the AMD GWAS from Fritsche et al. (2016), Figure S3. Categorization flow-chart of articles citing the AMD GWAS from Fritsche et al. (2013), Table S1. Articles citing the AMD GWAS from Fritsche et al. (2016), Table S2. Articles citing the AMD GWAS from Fritsche et al. (2013), Table S3. Regulated genes by AMD genetics in different studies, Table S4. Effects of AMD genetics on gene expression in AMD-associated loci, and Table S5. Effects of AMD genetics on gene expression in loci not associated with AMD to datei.
