# Supplementary material for: Learning from Fifteen Years of Genome-Wide Association Studies in Age-Related Macular Degeneration
Source: Cells. 2020 Oct 10;9(10):2267. doi: 10.3390/cells9102267 (PMC7650698; doi:10.3390/cells9102267)
Supplement: Supplementary file 1 [file cells-09-02267-s001.zip › Figure S1.pdf]

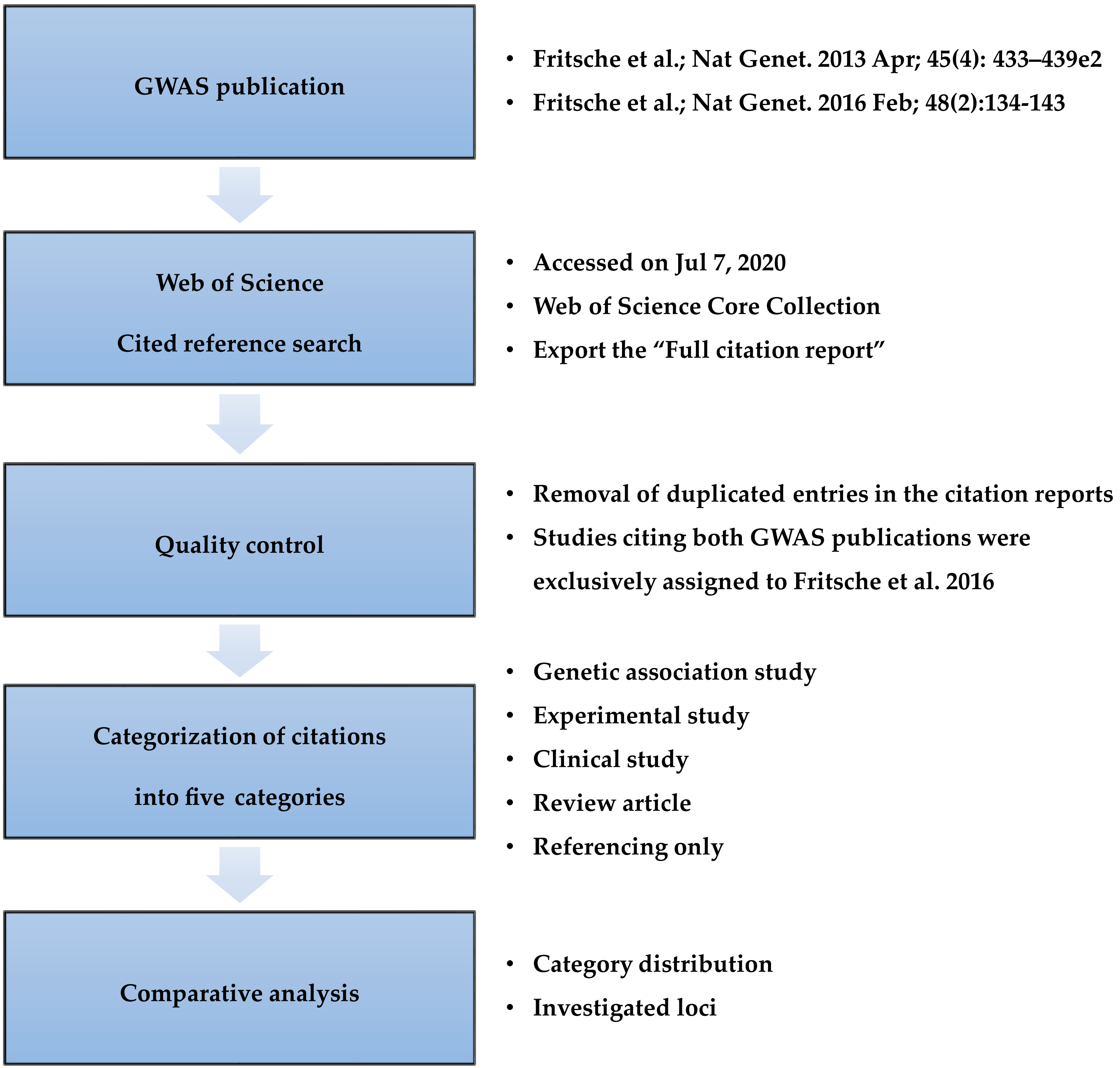
GWAS publication
Fritsche et al.; Nat Genet. 2013 Apr; 45(4): 433–439e2
Fritsche et al.; Nat Genet. 2016 Feb; 48(2):134-143
Web of Science
Cited reference search
Accessed on Jul 7, 2020
Web of Science Core Collection
Export the “Full citation report”
Quality control
Removal of duplicated entries in the citation reports
Studies citing both GWAS publications were exclusively assigned to Fritsche et al. 2016
Categorization of citations
into five categories
Genetic association study
Experimental study
Clinical study
Review article
Referencing only
Comparative analysis
Category distribution
Investigated loci
