# Supplementary figures and images for: Learning from Fifteen Years of Genome-Wide Association Studies in Age-Related Macular Degeneration
Source: Cells. 2020 Oct 10;9(10):2267. doi: 10.3390/cells9102267 (PMC7650698; doi:10.3390/cells9102267)

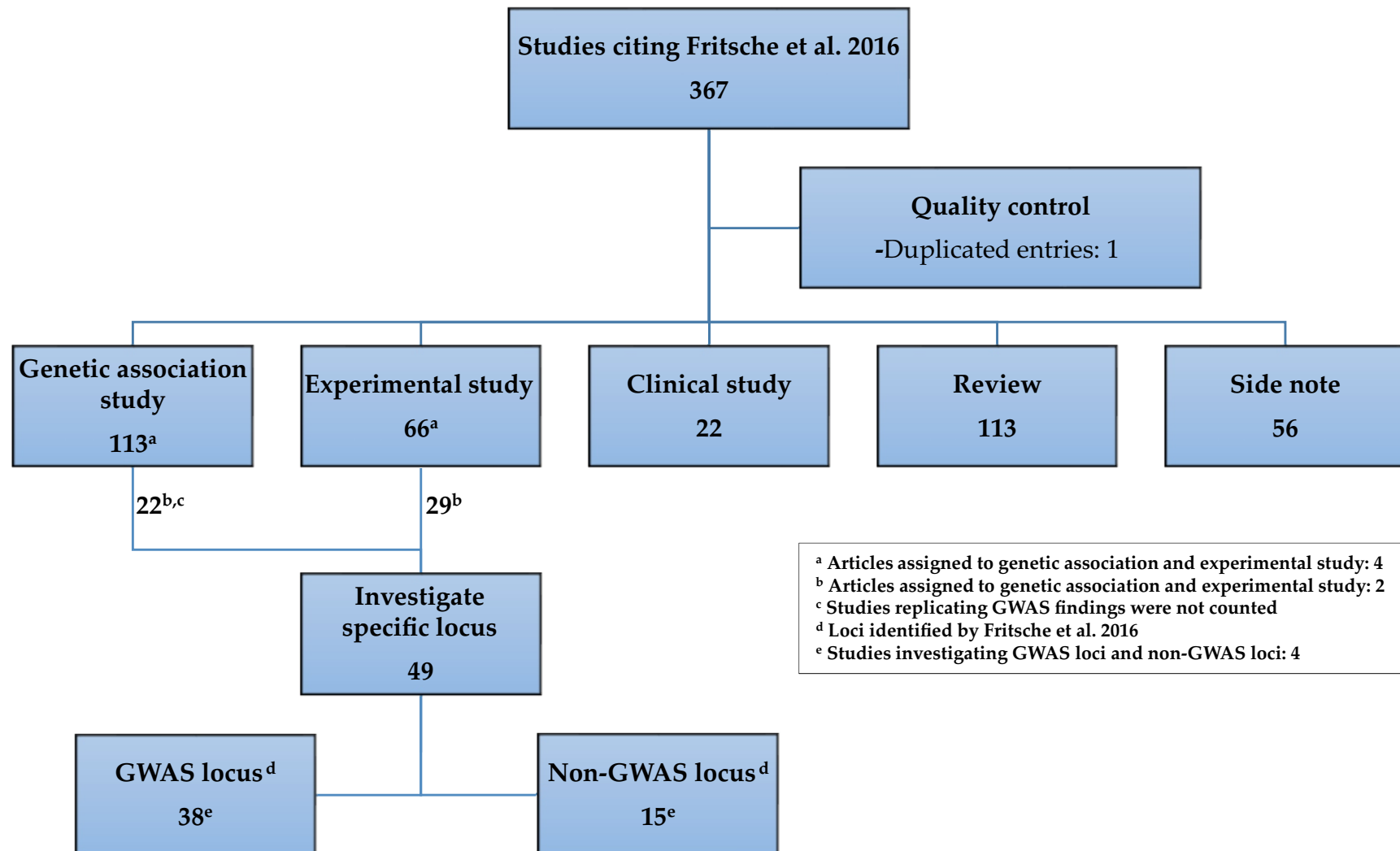

Supplement: Supplementary file 1 [file cells-09-02267-s001.zip › Figure S2.pdf]

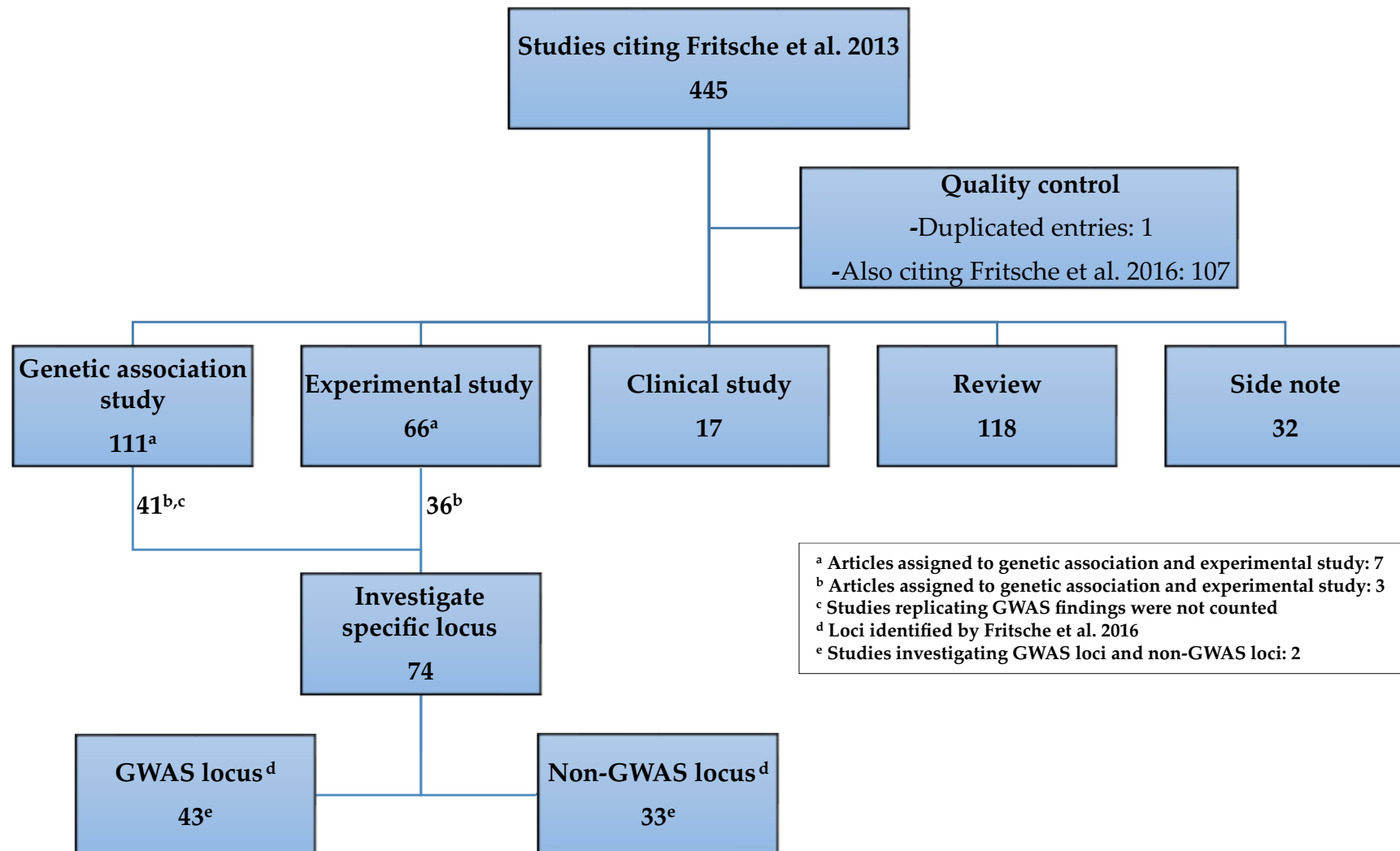

Supplement: Supplementary file 1 [file cells-09-02267-s001.zip › Figure S3.pdf]
